# “Dem sey mi mad”: a scoping review of the attitudes and beliefs of English-speaking Afro-Caribbeans about psychosis

**DOI:** 10.3389/fpsyt.2024.1385525

**Published:** 2024-08-19

**Authors:** Sommer Knight, Xin Qiang Yang, G. Eric Jarvis

**Affiliations:** ^1^ School of Psychology, University of Ottawa, Ottawa, ON, Canada; ^2^ Department of Psychiatry, McGill University, Montreal, QC, Canada; ^3^ Culture and Mental Health Research Unit, Institute of Community and Family Psychiatry, Jewish General Hospital, Montreal, QC, Canada; ^4^ Division of Social and Transcultural Psychiatry, McGill University, Montreal, QC, Canada

**Keywords:** psychosis, Afro-Caribbean, ethnicity, culture, psychiatry, racism

## Abstract

**Introduction:**

The mental health disparities suffered by the English-speaking Afro-Caribbean diaspora living with psychosis in North America and the United Kingdom have been well described for decades, but the root causes of these disparities remain poorly understood. Part of the problem may be that the attitudes and beliefs of Caribbean communities regarding psychosis have never been systematically assessed. Such an inquiry could lay the foundation for changes to how psychiatric services for psychosis are implemented with migrant Caribbean communities. The ideal would be a re-design of services, or cultural adaptation of care, based on input from community members, patients, and their families, with the hope that disparities of care would be reduced or eliminated as clinicians co-create interventions that are more appropriate and acceptable to Caribbean people. To lay the groundwork of such an important endeavor, we investigated the shared attitudes, beliefs, experiences, practices, and traditions of English-speaking Afro-Caribbean people in relation to psychosis and psychiatric care.

**Methods:**

We conducted a scoping review by searching Medline, PsychINFO and Scopus, reviewing 764 articles, and selecting 220 for thematic content analysis.

**Results:**

We highlighted the heterogeneity in the Caribbean diaspora living in North America and the UK. Five principal themes emerged: (1) The enduring effects of colonialism on the psychiatric care of Afro-Caribbean migrants; (2) The effects of adaptation to migration on the experience of psychosis; (3) Pervasive cultural mistrust of psychiatry and mental health institutions; (4) A collective approach to life; and (5) The role of religion and spirituality in the understanding of psychosis.

**Conclusion:**

Historical, sociocultural, and geopolitical themes characterize the English Afro-Caribbean experience of psychosis and inform culturally adapted clinical interventions for patients with psychosis and their families. Careful attention to these adaptations will reduce clinical bias and misdiagnosis, optimize adherence to treatment, engage patients and families in recovery, and ultimately, reduce treatment disparities while empowering Afro-Caribbean people and their communities. By bringing forward the themes in this chapter, individual clinicians will be given tools to change how they work with Caribbean people with psychosis in addition to laying the foundation for higher order changes in the mental health professions and society as a whole.

## Introduction

Schizophrenia spectrum and other psychotic disorders are characterized by delusions, hallucinations, disorganized thinking and speech, grossly disorganized or abnormal motor behavior and negative symptoms (1). Schizophrenia is a severe psychotic disorder with significant functional impairment that affects at least 20 million individuals worldwide, according to conservative estimates (2). In Canada, 1% of the population lives with schizophrenia, and the all-cause mortality rate of these Canadians is 2.8 times higher than people without the diagnosis: schizophrenia accounts for significant individual suffering as well as socio-economic burden (3).

### Definitions

This chapter will refer to notions of culture and ethnicity (4). *Culture* refers to implicit and often taken-for-granted beliefs, expectations, traditions, and patterns of thought and behavior that for an individual may appear to arise spontaneously from their thoughts and actions but on closer examination represent transmitted knowledge from previous generations and from the surrounding social world. Culture may be shared by ethnic, linguistic, or religious groups and communities, but may also be shared by members of professional orders and institutions of society, which may exert powerful effects on mainstream structural norms and practices. *Ethnicity* refers to groups or communities with shared history, language, religion, geography, and cultural practices that mark them off from other groups. *Race* refers to groups identified by superficial physical characteristics with a presumed biological or genetic origin but that are principally social designations with social consequences. *Racialized* communities refer to identities based on race that arise from historical ties to oppression and discrimination (4). All these categories impact Afro-Caribbean migrants as they adjust to life in systemically racist societies in North America and Western Europe.

### The role of culture in schizophrenia and psychotic disorders

Psychiatry as a medical speciality began in 19^th^ century Europe, therefore much of its concepts, categories, theories, and practices were based on the European experience. Mental disorders in the European context came to represent psychopathological norms, with which all other cultures were compared (5). Diagnostic classifications, like the Diagnostic and Statistical Manual of Mental Disorders (DSM) and International Classification of Diseases (ICD), are historically rooted in European paradigms of mental health and illness (6). Tools and interventions developed in Europe and North America may not readily apply to members of other groups, whose beliefs about mental illness and health may differ from European standards (7, 8). This can lead to mental health disparities in culturally diverse populations, especially when ethnic minorities also experience social disadvantage (9–13).

The specifics about the interactions between culture and mental illness are the focus of wide-ranging studies (14). Schizophrenia is incompletely understood, and many theories have been advanced by psychiatric researchers as to its etiology (15). Central to the current understanding of psychotic disorders is their multifactorial nature. The ethnocentrism of decades past has given way to exploration of cultural origins and experiences as contributing factors (amongst many) determining onset, expression and outcome of psychotic disorders (15). Furthermore, culture can influence the illness course and prognosis, with culture-specific explanatory models and responses to illness, and different responses to the illness (16, 17). These cultural differences exert a significant impact on health behaviors and clinical outcomes (18). There is urgent need to take into account the cultural context of patients and families and adapt the clinical approach accordingly, especially considering the importance of early intervention in psychotic illness (19).

### Background and history of the Afro-Caribbean people

The Caribbean, or the West Indies, is a geographical region of the Americas consisting of the Caribbean Sea, its islands, and surrounding coasts. Politically, a modern definition of the Caribbean is put forth by the United Nations geoscheme as a list of nation states (20). There is overlap and confusion between the terms “Caribbean” and “West Indies”. Their use has changed throughout history with no consensus on a single, unified definition of either term.

Although Indigenous populations lived in the Caribbean before colonization by Europeans, the region’s demographics changed dramatically with the arrival of slaves from West Africa in the 16^th^ to 19^th^ centuries, and with immigration from India, China, and Indonesia in the 19^th^ century. The Caribbean population is now predominantly of African origin, hence the term Afro-Caribbean, with significant sub-populations, including mixed origins, identified and named according to local traditions. European languages like Spanish, French, English, and Dutch continue to be spoken and are often the official languages of Caribbean nations; however, creole languages, which include Haitian creole and Papiamento, among others, are spoken as well. Christianity and its various denominations is the largest religion in the Caribbean (21).

The history of the Caribbean was transformed by European imperialism, colonization, and the Transatlantic Slave Trade (21, 22). Independence movements began as early as the Haitian revolution at the turn of the 19^th^ century, with various countries gaining independence and some remaining European colonies today (21). The modern era has been punctuated by intervention by the United States (US) and varying degrees of political turmoil (21).

The Caribbean is characterized by emigration and a sizable diaspora primarily residing in the United States, Canada and the United Kingdom (UK), with estimates of a one-to-one ratio of Afro-Caribbeans living abroad to residing in the region (23, 24). In Canada, Caribbean immigration was comparatively limited in numbers before the 20^th^ century. Afro-Caribbeans arrived in Canada as slaves, runaway slaves and laborers, but in relatively small numbers (22, 25). The years 1900 to 1960 constitute the first wave of immigration, with a first peak during World War I when laborers were recruited to work in mines (26). After 1945, women from Jamaica and Barbados began arriving in Canada as domestic workers under the West-Indian Domestic Scheme, which was in response to the postwar economic expansion (22, 25). This second wave lasted from 1960 to 1971, corresponding to liberal reforms of Canadian immigration laws (22, 25). The third wave continues to the current day in a climate of multiculturalism and ongoing labor needs in Canada (22, 25).

Currently, Jamaicans form the largest group of Canadians with Caribbean origins, followed by Haitians (mostly in Montreal), Guyanese, and those from Trinidad and Tobago (22, 25, 27). Indo- and Afro-Caribbean cultures are distinct as manifested by migration experiences, distinct lineages and heritages, family structures, and religious practices (28). Most Caribbean migrants settle in urban centers, with English-speaking Afro-Caribbeans settling in Toronto (province of Ontario), and French-speaking Haitians traditionally choosing Montreal (province of Quebec) (22, 25, 27). Nevertheless, there is a sizable English-speaking Afro-Caribbean community in Montreal (29).

The diaspora elsewhere in the world faces different policies than Canada’s official position of multiculturalism (30–32). The full complexity of the region and its people living locally and abroad needs to be acknowledged, but is beyond the scope of this review. Walker summarizes the diaspora’s mosaic nature in the following way:

“Even within any specific West Indian territory there exists a medley of ethnic types and heritages [ … ] to these ethnic differences must be added distinctions of class and occupation, education, urban and rural background, and historical experience. But even if the term ‘West Indian’ is imprecise […], it is still possible to identify a set of common features.” (22, p.3).

This scoping review will focus on English-speaking Afro-Caribbeans, which includes people from the islands of Jamaica, Barbados, Bermuda, Trinidad, St. Vincent, Grenada, among others, and the countries of Belize in Central America and Guyana in South America.

### Mental health disparities suffered by Afro-Caribbeans in the UK, US, and Canada

Disparities and inequalities in Afro-Caribbean mental health care, especially with respect to psychosis, continue to exist in the UK, US, and Canada. Research in the UK reports that patients of Afro-Caribbean background have high rates of schizophrenia and lower rates of affective disorders, especially depression, when compared to native-born White Britons (33). The Aetiology and Ethnicity in Schizophrenia and Other Psychoses (AESOP) study (34) systematically examined ethnic differences in illness characteristics and etiological factors of psychosis in the UK; it essentially confirmed older findings that Afro-Caribbean people were particularly vulnerable to psychosis. More recent studies have found not only high rates of psychosis in Afro-Caribbean and other migrants to the UK but similar findings elsewhere in Europe (35–38). One possible explanation for the greater risk of psychosis in Afro-Caribbean migrants is the community’s greater exposure to social disadvantages, including discrimination (39, 40), although some have questioned the presence of diagnostic error due to cultural bias in making the diagnosis (41). Other studies in the UK have examined the caregiver perspective and found that Caribbean families are not as involved in patient care, underutilize mental health services and experience added burden in supporting loved ones due to poverty, associated stigma, and distrust of services (42, 43).

More broadly in Europe, the European Network of National Schizophrenia Networks Studying Gene–Environment Interactions (EU-GEI) study showed that rates of psychotic disorders were elevated in racialized or ethnic minority groups (35) and specifically, in Afro-Caribbean people (38). A meta-analysis on migration and risk of psychotic disorders, consisting mostly of European studies, found higher risk in migrants including Afro-Caribbeans (37). Exposure to systemic risks, like threat, hostility, and violence, could contribute to the higher risk of psychosis in migrants (36).

Although not an epidemiological study, the National Survey of American Life (NSAL) investigated the mental health of the Black American population overall, and specifically studied Afro-Caribbeans as a standalone group and their experiences with psychotic illness and mental healthcare (6). For instance, a link was made between mental health risks, including psychosis, and the increased societal stress and downward social mobility associated with migrating from the Caribbean to America (44). Underutilization of services by Afro-Caribbean Americans was also reported (45), which may suggest that socioeconomic inequalities may, at least in part, explain the differences in help-seeking behaviors related to mental health (46). Robinson et al. found that Black Caribbeans in the US reported more depression than other Black Americans (47). Generally, however, the US data on psychosis in African Americans does a poor job distinguishing between those who were born in the United States and those who migrated from Africa or the Caribbean. Many studies have found that Black Americans are diagnosed with schizophrenia and psychotic disorders at greater rates than White Americans with similar symptoms. The most common reason invoked to explain these differences in diagnostic pattern is clinician bias that leads to misdiagnosis (48), although articles since the deaths of Breonna Taylor and George Floyd have been interpreting these findings to be more related to systemic racism in mental health care (49).

In contrast, there have been no such studies in Canada, where relevant data remain scattered (50). A scoping review in 2015 found 26 studies on the role of culture in mental health and service utilization among Canadian immigrants, of which 4 touched upon Afro-Caribbeans but none on psychosis specifically (51). There is, however, evidence that disparities or inequalities in the management of psychotic disorders exist in Canada as well. For instance, differences in pathways to care were found between people of Afro-Caribbean and European origins: Afro-Caribbean patients were more frequently brought to emergency psychiatric services by police, in the cities of Toronto and Hamilton (52). Another study demonstrated a positive correlation between being Afro-Canadian and police or ambulance referral to emergency psychiatric services in Montreal (53), although the distinction was not made between Afro-Canadians of different origins (e.g. being Afro-Caribbean) due to small study numbers. A later study in Toronto attempted to identify psychosocial stressors leading to presentation to emergency psychiatric services, and potential variations between ethnocultural groups. Economic and housing factors were the leading stressors for Afro-Caribbean patients (54). Studies have also examined compulsory detention. Canadian results echo international findings that Black patients (including Afro-Caribbeans) are more often admitted to psychiatric care against their will (55, 56). A study in Montreal found Black patients with first episode psychosis to have worse follow up than patients from other ethno-racial groups (Nikolitch et al., 2018). Other studies found that Black psychiatric inpatients were more likely to be diagnosed with psychosis than their White counterparts (57), and that Black Canadians were more likely to be depressed or anxious due to experienced discrimination (58, 59).

More generally in the Canadian context, conversations about the intersectionality of race, ethnicity and mental health are few but growing (60, 61). Recent political events like the *#BlackLivesMatter* movement and racial disparities from COVID-19 deaths (62) are stark reminders that Black people, including Afro-Caribbeans, continue to suffer from systemic racism in North America (63, 64). There is an ethical responsibility to investigate, understand and address these disparities. Individuals in society are cultural beings whose traditions, beliefs and values should be recognized and respected, especially in the field of mental health care, which aims to serve and promote the well-being of all (8).

### Laying the groundwork for care that is culturally adapted to Afro-Caribbeans

With the goal of improving psychosis care, services, and outcomes, one possible solution is modifying existing programs by culturally adapting them to the needs of the Afro-Caribbean community. If well implemented, such adapted interventions may improve outcomes. A recent systematic review and meta-analysis that included 46 culturally adapted interventions worldwide showed promising results (65). Cultural adaptations were grouped into different themes (including language, cultural concepts and illness models, family interventions, etc.) and showed significant reduction in symptom severity compared to non-adapted interventions (65). However, the same authors highlighted that the process of adaptation must consider local factors, like available human and material resources, health infrastructure, and the willingness to change in the dominant society. For instance, one review showed that in Canada, Black youth face several barriers to mental healthcare in spite of theoretically universal access (66). The responsibility for mental health care falls to provincial governments, resulting in remarkably different systems across provinces that are continually evolving. Furthermore, adaption must correspond to its intended population. There is limited research on adapting interventions for Afro-Caribbean families, with just one study of this kind in the UK demonstrating its feasibility with good rates of recruitment, attendance, retention and data completion (67). Cultural adaptations included specifically broaching discrimination and racism, being open to different models of care, and developing a shared learning approach. Clinicians need to be trained in practices like cultural humility to deliver culturally adapted interventions in safe and effective ways.

This review will proceed by asking: what are the shared attitudes, beliefs, experiences, practices, and traditions of English-speaking Afro-Caribbean people in relation to psychosis and psychiatric care? The answers will lay the foundations of a more inclusive, equitable and relevant approach for researchers and clinicians when engaging Afro-Caribbean patients with psychosis and their families. We complement our review with a clinical case vignette, to illustrate our findings with an example from daily practice.

## Method

### Study design

Scoping review is an approach to evidence synthesis that determines the scope and focus of the literature on a given topic (68, 69). This is ideal for the purpose of our study as we ask broad questions and survey past work to guide and organize future research interests and ideas. In our reporting, we adhere to the PRISMA for scoping reviews where applicable (70).

### Search strategy, article selection and data extraction

To begin, a librarian conducted an electronic database search. The databases Medline, PsychINFO and Scopus, were searched using MeSH concepts and keywords in title and author keyword fields. Search terms included psychotic disorders; psychosis; schizophrenia; mental illness; mental health; health knowledge; religion; spirituality; belief; attitude to health; attitude to illness; explanatory model; causal attributions; faith healing; traditional medicine; religion; spirituality; hallucination; delusion; madness; spirits; African; Caribbean; and Black (c. f. **Appendix** for search strategy). Additional papers from the grey literature, such as conference presentations and material from Black community organizations, were reviewed. Acceptable articles were included to identify and integrate Afro-Caribbean attitudes and beliefs that may not be highlighted in mainstream sources or found through traditional peer-reviewed research.

Two of the authors (SK, XQY) screened the selected articles to identify those relevant to Afro-Caribbean populations. All articles were stored using Zotero. Articles were rated from 0–2. 0=not relevant (these were excluded), 1=some information of interest, and 2=directly relevant to our search.

The search was conducted only for English-speaking Caribbean countries. Articles solely evaluating Haitians (French-Creole), Dominicans (Spanish), or people from other language groups were not included in this study. Articles were excluded if they were not written in English, if they were duplicates, or if the full texts were inaccessible. We included articles of various methodologies. Any uncertainties or disagreements related to the relevance of articles were resolved via discussion among the authors.

**Figure 1:**
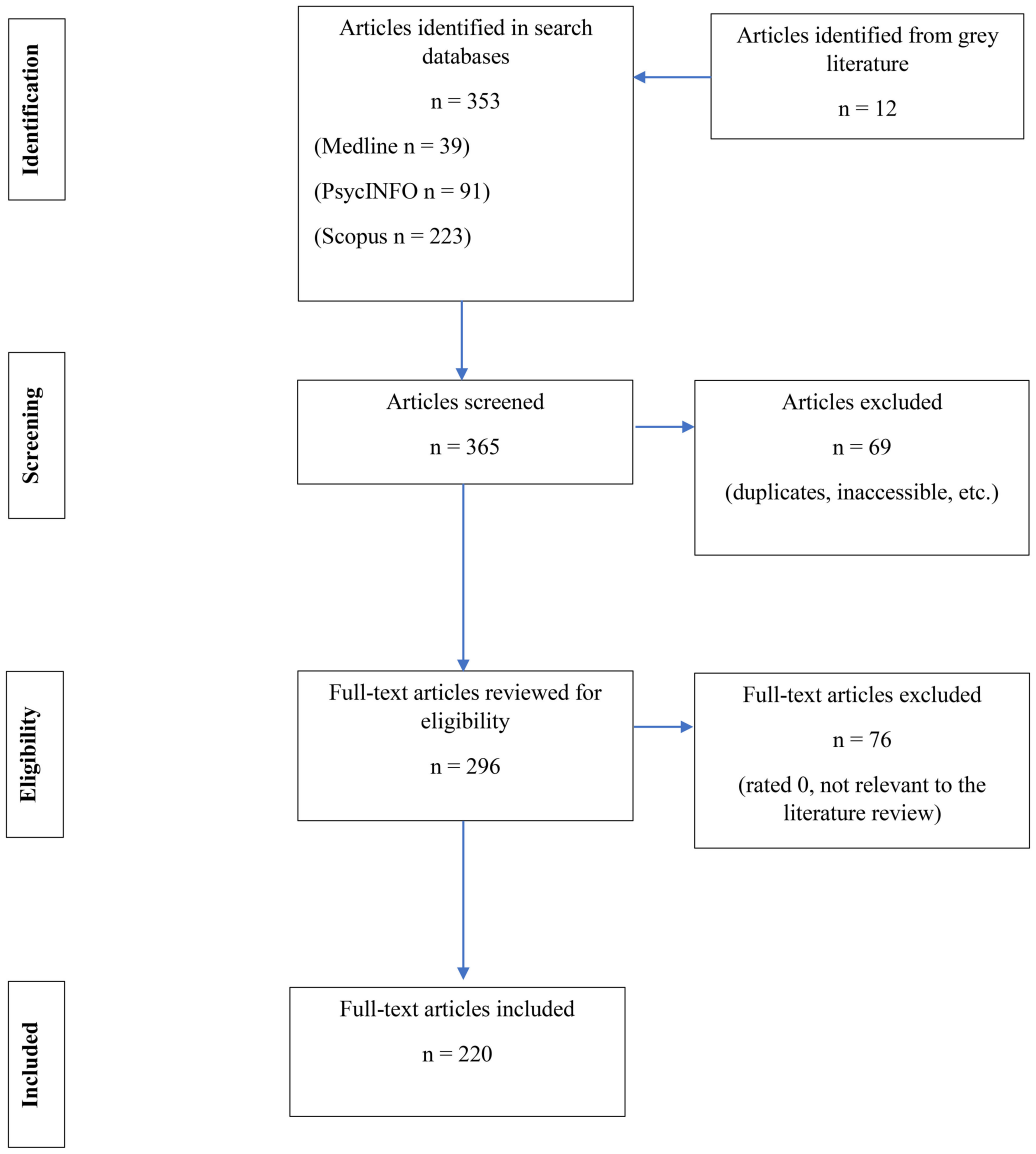
PRISMA Flow diagram.

A total of 296 articles were reviewed after Zotero identified and removed duplicates (see **Figure 1**). Of these, 76 articles were excluded and 220 remained for assessment. A data extraction spreadsheet was created in Microsoft Excel with pertinent characteristics of eligible studies (i.e. type of study, location of study, and study participants). All eligible articles were extracted from the Zotero database (c. f. **Appendix** for rest of references included in our study, as not all were directly cited in text).

### Analysis of English-speaking Caribbean attitudes and beliefs regarding psychosis

Data was analyzed inductively using thematic content analysis (71, 72). Using the extracted and summarized data of attitudes and belief systems, we generated emergent themes and subthemes. The following steps were taken: (1) Read the text line-by-line numerous times (familiarization); (2) Generate initial codes on English-speaking Afro-Caribbean culture from relevant notes; (3) Identify emergent themes related to beliefs and values in the data and categorize them based on conceptual similarities; (4) Review emergent themes; (5) Define and name themes and subthemes; and (6) Complete the write-up. The analysis was primarily conducted by the first author, who read the retained articles in their entirety; the second author and third (senior) author read seminal articles, key parts of other references; the team met and debriefed frequently to scrutinize the methods and results iteratively.

We used data from a variety of sources and disciplines. We welcomed input by discussing our progress with other teams who were reviewing similar research questions for other ethnic groups. We assessed contextual factors, like the researcher’s backgrounds, personal experiences and interest, and reflected on how they could have influenced the review (which we make explicit in our positionality statement).

### Positionality statement

Before presenting findings, we acknowledge our standpoints as people educated and working in Canadian academic institutions. The first author is a Jamaican-Canadian, second-generation immigrant woman, born and raised in a lone-parent household with a research focus on intersectionality, mental health disparities and culture. She is currently a doctoral student in Clinical Psychology at the time of writing. The second author describes himself as born in China, raised in Montreal, Canada, of a middle-class family, a cis-gender heterosexual able-bodied man, with medical training and at time of writing, a psychiatry resident, with interest in diverse conceptualizations of mental health and illness beyond the main curriculum. The senior author was born in the United States but has lived most of his life in Canada. He is of British, German and Danish ethnic origins and is a member of the Church of Jesus Christ of Latter-day Saints (Mormon). Professionally, he is a cultural psychiatrist and associate professor of psychiatry at McGill University and has been writing about the mental health effects of racism and discrimination for almost three decades. By adopting a self-reflexive stance, we humbly acknowledge that our positionality influenced our work. Through recognizing differences among the authors, exchanging with colleagues, and benefiting from the peer-review process, we were able to broaden our views and address potential bias.

## Results

### Afro-Caribbean attitudes and beliefs regarding psychosis

**Figure 2:**
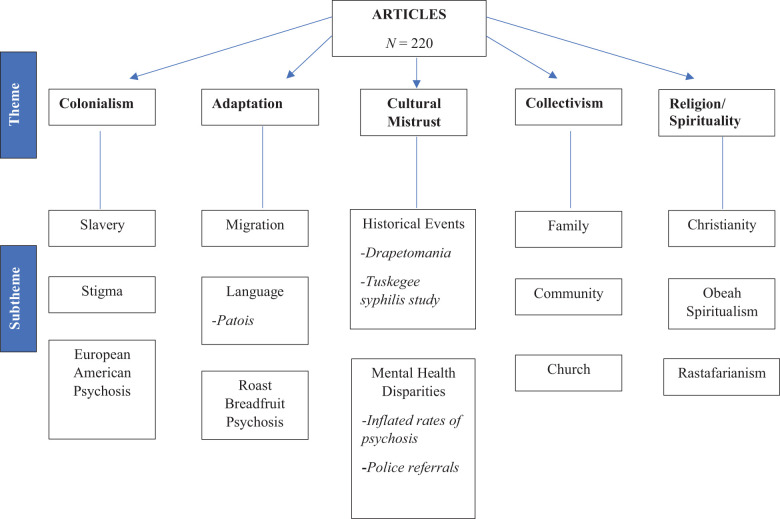
Emergent themes and subthemes regarding Caribbean attitudes and beliefs about psychosis.

Five core themes emerged from the literature review data: *colonialism, adaptation, cultural mistrust, collectivism*, and *religion and spirituality* (see **Figure 2**).

#### Theme one: colonialism

Caribbean culture derives from islands that are geographically close and share a history of colonization and enslavement (73). The slave trade led to a mixing of Black peoples in the Caribbean, so that despite the shared African origin, there is heterogeneity of culture, language, economic development, and educational opportunities among the various islands (74). This is evident in the Caribbean term “Creole” meaning the mixture of language and people of Black and European descent (75):

The intense historical relationship linking Jamaica and Britain to 300 years of the transatlantic slave trade and 200 years of colonialism has left 2.7 million souls living in Jamaica, 80% of African origin, 15% of mixed Creole background and 5% of Asian Indian, Chinese and European ancestry. With a per capita gross domestic product of US$4104 in 2007, one-third of the population is impoverished, the majority struggling for economic survival (76, p.9).

In response to European colonization, Caribbean peoples fought for political independence with some success from the 19^th^ century, however, European imperialism continues to influence the lives of Afro-Caribbean people today. Enslavement had harmful effects on the mental health of slaves:

The mortality rate of African slaves was the highest in the first century of slavery, and it was not unusual for reports of horrendous murders of African slaves inflicted as punishments by their masters. During slavery, the health of slaves was the responsibility of the slave owners. It is likely that violent mentally ill slaves in this period would be ‘put down’ by their owners [ … ] In the first half of the eighteenth century some slaves who were deemed to be mentally ill were incarcerated in plantation dungeons or ‘hot houses’. Jamaica’s first Lunatic Asylum was part of the Kingston Public Hospital, established in 1776. Involuntary commitment and custodialization were the principal tenets for the public policy provisions for the management of the violent, disturbed mentally ill in Jamaica and the West Indies (77, p.438).

In the mid-20^th^ century, British writers described Jamaican people as psychologically immature, and they were deemed inferior when compared to their White British counterparts (78, 79). Franz Fanon described the effect of these attitudes on the Black psyche and repudiated these racist European values:

In the twentieth century the Black man on his home territory is oblivious of the moment when his inferiority is determined by the Other. Naturally, we have had the opportunity to discuss the Black problem with friends and, less often, with African-Americans. Together we proclaimed loud and clear the equality of man in the world … And then we were given the occasion to confront the White gaze. An unusual weight descended on us. The real world robbed us of our share. In the White world, the man of color encounters difficulties… (80, p.90).

In Caribbean culture, some people believe that Europeans and North Americans are suffering from a collective delusion that their cultures are superior to those of racialized people worldwide and thus endorsing White supremacy:

Psychohistoriographic analysis proposed the concept of the European-American psychosis, which refers to the 500-year collective delusion of European world ownership and White supremacy based on Divine Right. The analysis identified a number of watershed periods in history, driven by the collective revolutionary struggles of colonized people of color worldwide, which systematically confronted the European imperative to own and colonize the world. These historical events include the violent and nonviolent resistance to slavery, emancipation from slavery, independence struggles, and the fall of apartheid as examples of the collective world encapsulations, or restrictive formations, of the European delusional system (79, p.21).

In this last excerpt, Hickling is not suggesting a literal psychotic disorder in White people but rather a group deception that justifies White supremacy at the expense of peoples of color around the world. Taken together, these quotations from the world literature describe the profound negative effects that colonialism and enslavement had on African peoples of the Caribbean. Indeed, Caribbean countries are still recovering from the trauma of centuries of violence, poverty, and subjugation. Living with these legacies is a daily stress and exhaustion for Black people and has chronic impacts on mental health and how Black people are viewed and treated by mental health care systems. They are linked indirectly to psychosis as the stressors that may predispose Black individuals to severe mental disorders.

#### Theme two: adaptation

When migrants arrive in a new country they must adapt to new languages, cultures, and customs (81). Many Afro-Caribbean people migrate from their homes to wealthy countries such as the United States, Canada, and the United Kingdom, to obtain a better quality of life for themselves and their families (82). The first government legislation that led to large-scale Afro-Caribbean migration to Canada was the West Indian Domestic Scheme, where the government invited Afro-Caribbean women to perform domestic labor for White Canadian women and their families (83, 84). In the 1960s, when the West Indian Domestic Scheme was in place, many Afro-Caribbean children were left behind as their mothers migrated to earn money with the hopes of later bringing their children to Canada (84–86). However, the migration experience was difficult for many people and required adaptation to various aspects of Canadian culture and setting, such as the climate, food, language, social norms, values, expectations, and sources of social support (87). As people began to migrate to Canada, some found that the host culture was not as welcoming as initially hoped, and the new migrants experienced discrimination and prejudice (85, 88). These kinds of post-migration stressors are especially important to consider when Afro-Caribbean youth are diagnosed with first episode psychosis:

If social adversity, in a variety of forms, particularly if experienced for prolonged periods during early development, increases the risk of psychosis, then it may be that this is 1 contributory explanation for the high rates of psychosis among African-Caribbeans and other migrant groups. It is known, for example, that migrants are more likely to settle in urban centers and are subject to a greater degree of social adversity in general than their indigenous [White British] counterparts (87, p.407).

The rigors of adapting to White norms after migration, whether in Canada or the UK, may produce in some cases a phenomenon observed in Caribbean communities termed “*roast breadfruit psychosis* … *whereby Black colonials could become and believe themselves ‘White’ by the integration of the coloniser’s language and culture into their own psyche in an attempt to completely assimilate the coloniser’s dictates”* (89). Once again, Hickling is not suggesting a bona fide psychotic disorder in these individuals but is alluding to the pressures on Black people to conform to and accept the norms of White society:

The roast breadfruit is a peculiarly, though not exclusively, Caribbean dish, and describes the cooked version of the breadfruit (Artocarpus altilis), which grows in profusion all over the Caribbean having been brought there from Malaya by transatlantic traders … when roasted, the breadfruit’s green skin becomes charcoal-black, while the inner flesh is White in color and doughy consistency with a similar taste to bread. Its characteristic appearance has aptly leant itself to the description of Black people who think themselves White (89, p.132).

#### Theme three: cultural mistrust

Cultural mistrust, a term expounded by Black clinical psychologist Arthur Whaley, refers to the lack of trust that people in the Black diaspora have towards White people and White institutions due to current and past experiences of oppression (90). Cultural mistrust is an adaptive behavior for Black people in racist and discriminatory settings:

Such findings are consistent with the argument that therapeutic context, reflecting the power relationships and cultural values of the larger society, may elicit cultural mistrust in African Americans … Thus, it is quite plausible that Black clients or patients exhibit paranoid-like behaviors during interracial therapeutic encounters … that low self-disclosure, which has been interpreted traditionally as a manifestation of psychopathology, may be due to cultural mistrust or adaptive paranoia. Thus, a healthy response to a racist society may be misinterpreted as pathology by mental health professionals (90, p.556).

A related and older concept, cultural paranoia, coined by Grier (91), refers to mistrustful behaviors that are more extreme than mistrust, arise from racist environments, and represent a necessary response to toxic surroundings:

For a Black man survival in America depends in large measure on the development of a “healthy” cultural paranoia. He must maintain a high degree of suspicion toward the motives of every White man and at the same time never allow this suspicion to impair his grasp of reality. It is a demanding requirement and not everyone can manage it with grace (91, p.161).

Part of the oppressive environment that gives rise to cultural mistrust includes medical mistreatment of African people of which several instances were mentioned in the reviewed papers. In 19^th^ century Southern United States, some Black slaves were diagnosed with “drapetomania”, a state of alleged madness causing slaves to run away from their masters:

Cartwright seemed unable to consider any other explanation for a slave’s wish to flee captivity. To him, slavery was the natural state of African Americans because he considered them to be like children and in need of constant care. For a slave to abandon her master was such a ridiculous notion to Cartwright, and the act so desperate, that he created Drapetomania to explain the behavior in a manner consonant with his political and economic interests (92, p.230).

In the 20^th^ century, African American men were exposed to unethical experiments in Tuskegee to determine the natural course of Syphilis and were not given treatment for the illness, even when treatment options became available: “We find that the disclosure of the study in 1972 is correlated with increases in medical mistrust and mortality and decreases in both outpatient and inpatient physician interactions for older Black men” (93). The overdiagnosis of schizophrenia also became of special importance during the civil rights movement in the 1960s, when African Americans, particularly men, were admitted to asylums for supposedly delusional thinking that in hindsight was linked to protest of the status quo. “From the 1960s onward, patients described by doctors as African American, paranoid, delusional, and violent had disproportionally high chances of being diagnosed with schizophrenia” (94). These examples highlight the strong and enduring relationship between being of African origin and misdiagnosis of psychosis, which antedated the 1960s by more than 100 years (92, p.230).

#### Theme four: collectivism

In Caribbean culture, it is important for everyone to meet social expectations, and there is a high level of familial responsibility, especially if a loved one is sick. That is why, even when family members are living abroad, many will send barrels back home filled with necessities such as clothing and food to help their family:

African Caribbean women utilized their extended family networks to maintain their families abroad. Older Caribbean women, usually grandmothers and aunts, nurture, and care for displaced children, becoming carriers of culture as other-mothers or fosters mothers, while their female relatives or friends work abroad in order to send money and goods to their households back home. Communal mothering is crucial to the survival of kin members. My paternal grandmother helped raise my two cousins during the ten-year absence of their mother who had migrated to the United States for work (86, p.107).

The family disruption of these parental migrations is real. For years, children may have only sporadic contact with their mothers. Lindo describes one adaptation to this difficult reality:

I am investigating what is referred to as the “barrel children syndrome” within Caribbean culture. The term is used to describe children who have been “left behind” by one or both parents who have migrated. Transforming discarded used shipping barrels into both cultural and sculptural objects [ … ] The suspended barrels become channels through which these relationships and experiences can be re-enacted and reflected upon (95, p.56).

Familial responsibility is highly regarded such that in some Caribbean cultures it is believed that family ancestors and those who have died remain in contact with them in the form of a ghost or spirit. This ghost or spirit is referred to as “duppy” in Jamaican patois. “A duppy, according to African Caribbean belief, is the spirit of someone who has died, whose time has passed, it remains an active (often characterized as malevolent and mischievous) and forceful actor in the present.” (96) These ties to family and to spirits of ancestors could affect the diagnosis of psychosis and the treatment in family context.

#### Theme five: religion and spirituality

Religion is important in the Caribbean islands. Most people feel connected to God and religious communities, yet spiritual beliefs and practices vary within families (97). Religious diversity is intense, with Christian denominations being the most common (98). “The prevailing religion is Protestant, although the presence of African retentions such as Obeah and Pocomania are still widely and profoundly experienced, and the powerful Rastafarian movement emerged as a countercultural religious force after 1930.” (76) Rastafarianism originated as a political movement for the liberation of the descendants of African slaves: “Rastafarianism is at once a religion, reflecting theistic ideas and sacred rituals, and a political movement, articulating goals and objectives developed in the context of the socio-economic realities of life for the Black underclass in Jamaica.” (99) Heavy drug use among Rastafarians and other Jamaicans, including ganja (also known as marijuana), is seen as a cause of severe mental illness in Jamaica (7, 100, 101): “Many Rastafari were involved with the growing of ganja (marijuana) and became the target of the security forces under orders from the Jamaican government working with the United States government to destroy the ganja trade.” (99).

In the French-speaking Caribbean, *Voodoo* (also written as vodou, vodoun and vaudon) and *Obeah*, sometimes known as black magic, are practiced (102–104). In the Caribbean, magic was used as a political and social tool during the period of colonization and slavery to obtain liberation. In the French Caribbean, Haitians practised voodoo during the Haitian revolution to break free of France. Voodoo’s ritual magic involved making a pact or oath with African deities to bring about a favorable outcome (105): “The Haitian Revolution was rooted in the commonality of religious and cultural practices centered on Vodou, and its beginnings were marked by a pact between the revolutionary leaders and the Vodou laws or spirits…” (103).

In the English Caribbean, Obeah is a spiritual practice akin to Voodoo in which a curse may be put on others who are despised or coveted. Jealousy, in Obeah tradition, is referred to as “red eye” (8):

Obeah is an interlocking system of beliefs and practices for harnessing supernatural forces and sprits designed to affect non-human forces in an effort to cope with personal, physical, spiritual, and psychological distress … Obeah is also used to address health or illness, to harm or guard against real or perceived enemies, and to bring prosperity in academics, employment, love, politics, or life’s pursuits (106, p.83).

Obeah took root during the enslavement of West Indian people by the British and was a hoped-for vehicle to freedom (107). Those who frequently practice and believe in Obeah spirits are recognized as “warners”:

In some instances, individuals are recognized as being a “Warner”- a person who publicly predicts future events and ‘‘warns individuals and societies’’ of spiritual and social transgression. ‘‘Warners’’ publicly preach predictions of the future, while otherwise leading healthy, remarkably ‘‘normal’’ lives. The Warner or other devout believer in obeah spiritualism is culturally recognizable and distinguishable from the individual with schizophrenia who has fixed false delusional beliefs in the effects of obeah and is usually recognized by others and labeled culturally as being ‘‘mad.’’ (8, p.232).

The passionate religious life and creativity in the Caribbean and among Caribbean peoples, and the unfamiliar mannerisms and religious references, could predispose to misdiagnosis of psychosis by unaware clinicians.

### Case vignette and clinical reflections on the study themes

We adapt a case from McGill University’s Cultural Consultation Service, or CCS (4), inspired by real events, to illustrate the themes found by the scoping review and their clinical relevance. (See the Case Vignette **Box 1**).

Box 1 **Case Vignette**

A 21-year-old Afro-Caribbean man was referred to the Cultural Consultation Service for clarification of previous psychotic episodes. “I have temper tantrums when people vex me,” he says. Stressors include that his refugee application was deferred, he had been accused of assaulting police officers and had an upcoming court date, but he felt that being charged with a crime was in itself a form of assault. He was unable to find work, was unable to help his mother and younger brother financially (neither were accepted to Canada), his younger brother (3 years old) had new-onset diabetes, and he had two young sons but was separated from his spouse. The patient had a congenital neurologic disorder (with nystagmus) that gave him an odd demeanor during interviews. He saw an ophthalmologist once who recommended corrective eye surgery, but this scared the patient, so he never went back. Despite being referred with a diagnosis of psychosis, the patient did not have disorganized speech, paranoia, or delusions. Auditory hallucinations occurred intermittently and consisted of vague voices talking to him when he was “vexed.” They told him bad things about himself.

The patient felt sad much of the time, was frequently tearful, entertained suicidal Ideation (throwing himself from a building), was overcome by feelings of failure for not having provided for his family, and smoked Pot to go to sleep when he was sad or angry. He had an explosive temper which was triggered by being repeatedly bothered. He could punch his hand through a wall but denied ever harming people. He reported unfair treatment and repeated harassment by the police. On one occasion, he assaulted a police officer when he was stopped unnecessarily, and his head was slammed into a wall during the scuffle.

The patient came to Canada as a 14-year-old boy. He did not finish school but worked to support his family. He did not attend Protestant Church anymore because of what the people said about the unfashionable clothes he was wearing.

On examination, the patient was a short man with braided hair. Nystagmus was evident in both eyes. He made infrequent eye contact. Somewhat jerky body movements were noted. There was no thought disorder. The patient was calm and cooperative. His first language was a form of creolized English, so he was hard to understand sometimes. He spoke in a low voice and tended to mumble. He arrived one hour late, saying that he was mixed up by the bus system. He wept in the interview when discussing his family. As his voice choked with emotion, his girlfriend looked away and shook her head in sadness. He had suicidal thoughts. He expressed themes of discouragement about how difficult life had been in Canada. He was frustrated by the police who treated him harshly on several occasions. There was no delusional ideation and no wish to harm others. Perception was remarkable for intermittent, stress-related, and self-critical auditory hallucinations. Cognition was grossly within normal limits. Judgment was preserved during the evaluation. This patient presented less as someone with psychotic disorder than as a depressed man with overwhelming social adversity, limited support, and possibly an untreated neurological condition.

#### Case vignette and Theme 1: colonialism

The vignette illustrates how a young Afro-Caribbean man was separated from his family as part of the migration experience and could not find work to support his family financially, contributing to depression and poverty. One of the far-reaching consequences of colonialism and enslavement is that social and financial networks arising from colonial-era structures continue to disadvantage Afro-Caribbean people today. Clinicians must listen for these narratives and acknowledge their impact on Caribbean society, attitudes, cultural beliefs, and behaviors to appreciate the social disadvantage facing many Afro-Caribbean people and how living with disadvantage affects their mental health (73, 108). It should be noted that among Caribbean immigrants, those living in Canada become hubs of support and are expected to help family members after they arrive (109). In this man’s case, it seems that the aunt who welcomed him to Canada did not get along with him. He was forced to fend for himself and to become a hub for others before he had become sufficiently established.

#### Case vignette and Theme 2: adaptation

In the vignette, the patient was initially diagnosed with psychosis, without consideration of post-migration factors that may have impacted his mental health and well-being: racist interactions with police, crushing poverty, lack of opportunity, and misdiagnosis by those who were trying to help him. Psychotic disorders imply persistent symptoms like hallucinations, delusions, disorganized speech, disorganized behavior, and negative symptoms (e.g., lack of motivation, reduced emotional expression) rather than short-lived intoxicated or stress related states (110). The vignette provides a reminder that clinicians need to adopt a person-centered approach; in this case, the young man’s hallucinations did not indicate a psychotic disorder. Understanding the context of his difficult adaptation to life in Canada was crucial in making a diagnosis of depression, rather than psychosis. Instead of looking for the presence of symptoms that will confirm a diagnosis, clinicians need to develop a more complete, nuanced understanding of how symptoms arose, what role they served in the clinical context and the lifeworld of the individual.

Furthermore, linguistic issues also may complicate adjustment to life in Canada for some people from the English Caribbean because they are expected to speak English when, in fact, they grew up speaking Creole or Patois. Although these are English-based languages, they may be challenging for care providers to understand. This was the case for the patient in the vignette, who spoke an English creole. His non-standard English may have led to stereotypes about his credibility or intellectual capacity and could have added to the clinical impression of psychosis given the unfamiliar patterns of speech. To avoid culturally inappropriate diagnosis and treatment, clinicians must carefully consider cultural, linguistic, and religious issues in every case (111).

#### Case vignette and Theme 3: Cultural mistrust

Many Afro-Caribbeans share a lack of trust towards White people. Today, injustices persist as people with African ancestry are more likely to be involuntarily admitted to the hospital (55), report police contact on the way to emergency psychiatric services (52), report greater social coercion (112) and have poor follow-up for early psychosis compared to other ethnic groups (113). The patient in the vignette reported unfair and repeated harassment by the police. He expressed frustration towards the police who treated him harshly on several occasions, and even recalled an occasion in which he resisted the police when they stopped him unnecessarily. These kinds of experiences engender cultural mistrust – an expectation of discriminatory treatment and a compensatory wariness of interaction with the wider society. Clinicians may mistake cultural mistrust as paranoia and wrongfully diagnose Black people with psychosis. Furthermore, the lack of racial and ethnic minority representation in medical settings may exacerbate cultural mistrust when clinicians don’t share the same cultural concepts and language (42). These factors could easily have been at play for the man in the vignette, who was feeling isolated and unsupported by the wider society in the face of the adversity he was facing.

#### Case vignette and Theme 4: collectivism

Afro- Caribbean culture is collective and emphasizes the needs and desires of the family, the neighborhood, the church, and the wider community rather than those of the individual (114). There is a sense of interconnectedness and personal identity is often tied to one’s community (115) (Heinz et al., 2012). For these reasons, there is a strong family obligation for siblings to support their parents, for older siblings to care for their younger siblings, and for families to care for extended family members. This is highlighted in the clinical vignette where the young man was responsible for the welfare of his extended family, including mother and ill younger brother, who were not accepted to Canada. He felt deeply responsible for his family and their unresolved problems, and his inability to help them was at the root of his depression and distress. Given this understanding, clinicians should seek to support and empower the patient in his familial (and other) roles, including seeking collaboration with allied professionals and community resources.

#### Case vignette and Theme 5: religion and spirituality

Organized religion is central to the lives of many Afro-Caribbean people and most engage in religious services by choice, including adolescents (116). The vignette mentioned that the patient came to Canada at the age of 14 years and was attending a Protestant Church, but later left due to judgements by the members about his unfashionable clothing. How the patient experienced this change would need to be explored clinically (for instance, whether he understood it as a loss and would want to re-engage one day with the church). Understanding and treating the patient’s depression also would include an exploration of spiritual issues more generally and could be a productive therapeutic avenue. Additionally, the vignette mentioned the use of marijuana, so discussion about whether the use of marijuana is linked to Rastafari practices would be important. Given the frequency of religious and spiritual practices in people of Afro-Caribbean origin, clinicians need to examine individual context by obtaining religious and spiritual history from family or community. Questions to consider would include whether a patient’s practices have changed recently and to what extent they are within cultural norms. If needed, and with the authorization of the patient, clinicians can reach out to appropriate religious leaders for their input and support.

## Discussion

The aim of this scoping review was to explore the shared attitudes, beliefs, experiences, practices, and traditions of English-speaking Afro-Caribbean people in relation to psychosis and psychiatric care. Five themes emerged from the data: 1-*colonialism*, 2-*adaptation*, 3-*cultural mistrust*, 4-*collectivism*, and 5-*religion and spirituality*. These themes suggest the need for a person-centered approach to assessment and intervention for Afro-Caribbean patients and their families in which clinicians primarily seek to understand explanatory models of illness and meaning of symptoms. The themes imply that Caribbean patients feel misunderstood by their clinicians and that what is at stake to them is different from what their clinicians may consider important. Given these issues, the culturally sensitive and humble practitioner would not be in a rush to apply an official diagnosis, but diagnoses would be cautiously assigned to avoid premature and erroneous conclusions. The themes from this paper could act as a template or guide to clinicians when they are evaluating Afro-Caribbean patients with early psychosis and their families.

Regarding the first theme, colonialism, it is important for clinicians to consider that the psychiatric theories and practices that inform the diagnosis and treatment of psychosis are heavily influenced by Western beliefs (8). There is limited focus on the beliefs and practices of other cultures with the result that ethnic minorities find it daunting to navigate the mental health systems in White dominated wealthy countries like Canada, the US and the UK. Therefore, to decolonize clinical practice, cultural explanations of mental health and illness need to be more inclusive and accepting of Caribbean cultural knowledge with respect to psychosis. (117). Decolonizing psychiatry and psychology is a difficult and long-term process, but each practitioner can start locally with the patients they see by acknowledging the ongoing struggle with racism that Afro-Caribbean minorities face and the impact of historical colonialism on their mental health attitudes and expectations. Mental health professionals, despite clinical time pressures, need to find a place in their practices for decolonizing efforts. A systematic review conducted by Degnan and colleagues (65) showed that the greater the degree of cultural adaptation of clinical services, the more. effective the intervention will be. Training and workshops on culture and psychopathology, cultural competence and multiculturalism can de-center European practices in mental health care and guide clinicians away from misinterpreting culturally acceptable behaviors as symptoms of psychosis. Partnering with family members, church associates, and Caribbean community leaders in clinical services and seminars will reduce stigma and enhance therapeutic alliance (42). Given the history of asylums established by the British in Jamaica, clinicians should situate individual and family interventions outside hospital settings in churches or in homes, when appropriate. Encouraging family and community members to share personal stories and recovery narratives will also normalize family mental health experiences in cross-cultural context. Colonialism may seem to be a faraway problem in a distant time, but for Afro-Caribbean people, colonialism is surprisingly current and is at the root of many of the social inequities they experience daily. These stressors and issues contribute to poor outcomes for psychosis.

Adaptation is an important issue given how migration stories, and the need to adapt, have been so central to Caribbean peoples for centuries. Whether being forcibly transported to the islands from Africa in the distant past or uprooting voluntarily to relocate to Canada, the US, or the UK for economic reasons (118), Afro-Caribbean peoples have a long history of starting over and adapting to new settings and circumstances. Historic policies, such as the West Indian Domestic Scheme, have allowed for the large-scale migration of Afro-Caribbean people to Canada since the 1960s. However, many Afro-Caribbean migrants faced additional social and economic challenges due to the loss of social status after migration (118, 119). This adjustment becomes further challenging when a family member is diagnosed with a severe mental illness and families must learn to navigate an unfamiliar mental health system. Adapting to life in a new society and culture is a gradual process that can become overwhelming during such crises. Clinicians can reduce stress in these circumstances by employing linguistic interpreters and culture brokers to facilitate the sharing of key medical information and helping Afro-Caribbean patients and families understand and willingly participate in recommended interventions (120). Listening for cultural expressions with respect and curiosity will improve the therapeutic alliance and mutual understanding, while encouraging open discussion about family matters. **Table 1** shows examples of Caribbean cultural expressions with links to cultural explanations of illness and their roughly equivalent medical interpretations. Clinicians are encouraged to ask plenty of questions, give more time to evaluations of culturally different patients, refrain from making snap judgments, and be open to suggestions that come from Afro-Caribbean patients and their families (121). Clinical tools, like the Cultural Formulation Interview (CFI) and the Outline for the Cultural Formulation (OCF) published in the DSM-5-TR by the American Psychiatric Association, can guide clinical interviews with patient from diverse backgrounds and identify cultural factors influencing symptoms, suffering and treatments.

**Table 1:** Cultural expressions and concepts related to psychosis and mental illness in the English Caribbean.

Concept/Expression	Meaning	Interpretation
**“Yuh a idiat, yuh fool-fool”**	“You are acting stupid, like a fool”	Refers to someone who is not well and is speaking nonsense or behaving oddly particularly in the context of mental illness
**“Mi a tun fool-fool”**	“I am becoming foolish”	Refers to changes in cognition, behavior, and perception
**“Dem sey mi mad”**	“They say I am going mad”	Refers to others’ perception of mental/emotional health problemss
**“Mi a mad”/”Mi a go krazy”**	“I am going mad”/”I am going crazy”	Refers to changes in the person’s thinking, feelings and behaviors related to mental illness
**“Mi hed nuh good”**	“My head is not good”	Challenges with attention/concentration and memory
**“Mi nerv gaan”/”Mi nerv bad”**	“My nerves are gone”/”My nerves are bad”	Refers to feelings of anxiety
**“Mi a worry”**	“I am worrying”	Refers to generalized anxiety
**“A wa de pon mi head so?”**	“Why am I so worried/nervous?”	Refers to generalized anxiety
**“Mi nerve a go”**	“I am losing my nerves/mind”	Refers to severe anxiety or panic
**“Mi hav bad feelin”**	“I am feeling bad”	Refers to bodily or psychosomatic complaints; may be related to anxiety
**“Mi haunted”**	“I am haunted”	May be symptoms of paranoia, fear or anxiety
**Duppy**	Spirit	Refers to a spirit of someone who has died but is still actively present in one’s life
**“Duppy a ride mi”/”Ghost a falla mi”**	“Ghost is on me”/”Ghost is following me”	May be a spiritual experience or symptom of psychosis
**Duppy-lik**	Refers to a spiritual blow by an evil spirit	Occurs when someone has a “bad mind,” and they wish ill upon others due to envy
**“Bad spirit een a mi yaad”/”Mi a se fallin angel”**	“Evil spirits are in my home”/”I can see evil spirits”	May be a spiritual experience or symptom of psychosis
**Red Eye**	Obeah-based jealousy	Refers to someone who is jealous and uses Obeah black magic out of envy
**Warner**	Predictor/Fortune-Teller	Someone who can predict the future and warns others of events to come. *Note.* Warners can be distinguished from an individual with schizophrenia if they conform to spiritual beliefs or practices accepted by members of the family or community.
**Roast breadfruit psychosis**	Racial identity conflict	Refers to how Afro-Caribbean people who are visibly “Black”, act and behave “White” by adopting Euro-American mannerisms and language, while denouncing their Black identity and traditions
**European American psychosis**	European beliefs about their inherent superiority to other groups and cultures.	Refers to the belief that Europeans should own the world and that those who do not endorse European beliefs should be “othered” and converted to believe in European ideals. The pursuit of world domination and ownership by Europeans, and beliefs about their inherent superiority, are what some Caribbean people perceive to be mentally ill and delusional because these beliefs are rooted in fixed false beliefs and practices like racial discrimination

Adapted from Obeah-illness versus psychiatric entities among Jamaican immigrants: Cultural and clinical perspectives for psychiatric mental health professionals, by H. A. Ellis, 2015, Archives of psychiatric nursing, 29(2), p. 86. Copyright 2014 by the Elsevier Inc.

First episode psychosis programs adopt an early detection approach, with timely medication use, which aims to improve long-term outcomes. But there is much more to the treatment of early psychosis than medication. Over the treatment course, clinicians should pay close attention to enhance protective factors specific Afro-Caribbean people, who may lack a strong social support system in Canada. Mental health professionals can expand social networks by linking patients to community organizations, such as faith communities, by reaching out to local church leaders for support and suggestions about how to proceed. Community programs may reduce relapse rates for patients with first episode psychosis, like one established in Kingston, Jamaica (89), which employed a collective cultural therapy model of psychotherapy and was effective in helping members of Caribbean communities with serious mental health problems find psychological healing and build resilience (122). While implemented in Jamaica, Hickling’s model offers needed insight into the kinds of interventions that can be culturally adapted and what the form, process and content of successful programs may be. Hickling's adapted community programs incorporated cultural symbols, sociodramas, and art therapy to allow individuals lacking literacy to communicate and illustrate their dialogues and feelings (122, 123). This approach can also be especially helpful for patients and families who are new to Canadian languages and mannerisms. Visual aids are also another option to accommodate other forms of learning and information processing (42).

Cultural mistrust refers to the suspicion with which members of racialized communities view the institutions of White society (law, medicine, education, law enforcement, government). This suggests the need for mental health professionals to continuously consider the numerous sociocultural, historical, and political traumas that undermine access to mental health care for Afro-Caribbean people. Put simply, given the way that Afro-Caribbean people have been mistreated by white institutions for hundreds of years, it should not be surprising that many Black people feel uncomfortable or mistrustful of the society around them. In this light, cultural humility (124) is important for clinicians to better sympathize with the lived experience of Afro-Caribbean patients and their caregivers. There are numerous social and cultural determinants that influence the mental health of Afro-Caribbean migrants and that influence their sense of safety and security. By adopting a culturally humble stance, mental health service providers will reflect upon how discrimination is produced, maintained, and leads to mental health disparities, such as inflated rates of diagnosed psychosis (111), increased risk for police contact when psychotic (60), and greater rates of compulsory admission to psychiatry (125). Poor mental health outcomes in Afro-Caribbean populations are a consequence of the social disadvantages they face, which has sparked political movements like #BlackLivesMatter (126), and the fight against systemic racism that has gathered momentum in recent years (111). There is an inequitable distribution of resources, services, and opportunities for members of Black communities, and (111) these persistent inequities perpetuate cultural mistrust in Afro-Caribbean people.

To reduce cultural mistrust in Afro-Caribbean patients and families, clinicians can implement some simple changes to their routines. Group sessions should be co-led by Afro-Caribbean group facilitators, or faith and community leaders, to establish trust and build rapport (while being mindful of the added burden this may place on community partners, who should be compensated for their time). Medical administrators need to be mindful of hiring diverse staff for their clinical teams. Medication non-adherence is common among those diagnosed with first episode psychosis (127), and should be examined from a cultural lens among Afro-Caribbean groups with consideration for the impact of historical medical experimentation with the government’s approval and the effect of officially condoned abuses of Black communities on their trust of doctors, medication and pharmaceutical treatments. Clinicians should question their own biases that can cloud judgement, try to eliminate stereotypical images that they may have of Afro-Caribbean people and tailor treatment to match the needs of each patient and family (128–130). Clinical tools, such as the ADDRESSING framework put forth by Pamela Hays, through the American Psychological Association, can help clinicians reflect and acknowledge their privileges across different parts of their intersectionality, including but not limited to age, disability, religion, ethnicity and race (131). Given the heterogeneity among people from the Caribbean (74), clinicians should listen and attend to patient and family concerns by requesting the assistance of culture brokers or linguistic interpreters, when needed, and involving family members to learn about illness beliefs. These approaches will mitigate mistrust and foster open cooperation. Being aware that personal interpretations may be culturally out of sync with the social realities of Afro-Caribbean patients and families will promote cultural sensitivity and efforts to provide more patient and family centered care. Care providers can meaningfully work together and collaborate with family members and community partners to ensure that their views and expectations about the treatment are documented properly and can be readily accessed by the clinical team. Cultural mistrust is easy to ignore when working with Afro-Caribbean patients, but clinicians should have open discussions about how their patients view the hospital, their team, the police, and other aspects of their care. If mistrust is present, safe discussion may be a way to show understanding and genuine concern and invite a change of perspective.

Collectivism is fundamental to Afro-Caribbean life and refers to the relationships between people and the idea that family or group identities and goals supersede those of the individual. Smith and colleagues (132) found that Afro-Caribbean caregivers often attributed early psychosis to relationship issues, as opposed to biological or genetic causes rooted in the individual. Loyalty to family trumps other obligations, which is important in recovery from psychosis because the affected person always knows that family will be available to help and to support. The same family loyalty may also work against recovery in some situations when family matters remain private and are not voiced to outsiders, like members of the treatment team, out of fear of judgement and negative evaluation (74, 133). Despite collectivism as an overarching cultural value, negative attitudes towards severe mental illness, such as a psychosis, may induce fear, stigma, and avoidance from loved ones in some cases (134). Psychosis may undermine the collective values of Afro-Caribbean families and may overwhelm and confuse normal patterns of family function. When families have non-medical explanations of psychotic symptoms, the relationship with the treatment team may be compromised because clinicians may be assumed to be skeptical of alternative illness beliefs and ignorant of Black ways of handling distress. This can delay help-seeking or may reduce participation in treatment programs, with potential to worsen the patient's function and inhibit recovery. To encourage family involvement in early psychosis interventions with Afro-Caribbean patients, clinicians should offer informal home visits to family members, schedule additional sessions outside business hours, involve church or community leaders in joint meetings, and incorporate cultural beliefs into discussions so the material is more manageable for patients and their families. For some Black people, it is important for mental health professionals to establish a sense of normalcy for families by offering group forums with other Afro-Caribbean families, so family members know they are not alone. Other ways that mental health providers can enhance family involvement is by changing the environment in which the sessions take place. Meetings could be held outside of hospital settings and in a circular seating arrangement to represent interconnectedness and collective, shared values. Modifying standard approaches by introducing Caribbean practices and norms, such as traditional healers, sacred religious texts, and prayers, can catalyze the efficacy of the session for some participants (135).

Family members should also be encouraged to actively participate by giving them the opportunity to be involved in decision making and assessing not only the needs of the patient but of the family to reduce conflict and promote cohesion. Each family has distinct traditions, related to the larger Caribbean culture, that should be acknowledged and considered when implementing interventions for patients and their caregivers with psychosis. Given that medication non-adherence is a common issue, working with families can ensure that patients take the medication regularly. Treatment goals should also be realistic and aligned with Caribbean cultural values of collectivism to improve acceptability and support of the proposed interventions. There may be challenges in managing different cultural expectations among family members with different beliefs but reminding families and patients of the shared goal can help mitigate differences. Thus, collective values pervade Afro-Caribbean families and culture, with sometimes beneficial and sometimes problematic effects on the treatment alliance with patients. Clinicians need to reach out to families to offer education and practical advice that is specific to the cultural communities they are serving.

The final theme that emerged from the scoping review was religion and spirituality. Several religious practices and beliefs were identified in the reviewed papers, such as Obeah spiritualism, Christianity, and Rastafarianism. Giving due attention in the clinical encounter to religious beliefs may seem strange, or even uncomfortable, to many clinicians; but from the point of view of Caribbean culture and families, these are the kinds of interventions that may resolve the tension between European colonial ideals and Caribbean values (136, 137). Discussions about Christian spirituality and religion may open reflection about psychosis and its treatment and may reduce mistrust and augment a collaborative clinician-client relationship. Obeah spiritualism, on the other hand, and other forms of religious practice may be misinterpreted as delusion rather than a spiritual belief. Therefore, clinicians should check with culture brokers or family members to determine whether the patient’s behaviors conform to acceptable spiritual beliefs and practices (104). Family members are key allies to carefully unpack the complexities of these issues and prevent misunderstanding. Accepting to discuss cultural concepts, that make room for Caribbean beliefs in spiritual or supernatural agents, may reduce mental health stigma among family members and promote family unity. Diagnostic errors and the mislabeling of patient’s cultural behaviors can have direct treatment consequences for patients and their families in addition to undermining trust in mental health services. Mental health providers should openly ask Afro-Caribbean families about their religious beliefs to inform the treatment plan and provide common understanding between the clinical team and family. Inviting church officials and faith associates to follow up meetings (with the patient’s permission) may facilitate psychoeducation programs for Afro-Caribbean patients with psychosis and their families. Hickling and Paisley (8) found that a 33-year-old Black man, once diagnosed with schizophrenia in the US, was later deemed to be misdiagnosed and mislabeled upon his return to Jamaica. Obeah spiritual treatment was provided to alleviate the suffering that resulted from being wrongfully and involuntarily admitted to a psychiatric hospital and forced to receive antipsychotic medication after being brought to emergency services by the police. These issues highlight the need to consider each patient’s heritage and spiritual beliefs as a necessary component of person-centered culturally competent care. Although the religious and spiritual tapestry in the Caribbean is complex and mixed, mental health providers owe it to their patients to evaluate the role of religious beliefs and practices empathically and critically in the diagnosis and management of psychosis to reduce bias, improve the therapeutic alliance, and prevent misdiagnosis (138).

## Limitations and future directions

This study bears limitations intrinsic to a scoping review, including but not limited to the lack of structured appraisal of the quality of evidence. This review delineates the broad areas and divisions of studies already published on the topics of interest, which will hopefully inspire narrower, more specific research questions better answered through systematic reviews, or by original research. Several gaps in the literature can be attributed, at least in part, to the heterogeneity of the Afro-Caribbean community. Mental health is often at the intersection of important social, cultural, and political issues, like migration, poverty, women’s rights, LGBTQ+ activism, friction and conflict with the justice system, homelessness, and the special considerations of youth and elderly. Discrimination, in forms other than racism, certainly merit continued attention in future research, especially given the paucity of data and evidence specific to people of Afro-Caribbean heritage and the interaction of intersectional characteristics with first episode psychosis. This work has yet to be done.

More research is needed to understand the social and cultural suffering of this group to promote novel approaches and culturally adapted care. A deep-rooted history, that dates to enslavement, continues to impact the lives of Afro-Caribbean migrants, and this must be recognized and acknowledged in clinical settings. Furthermore, these groups experience additional discrimination and racial prejudice that exacerbate psychotic symptoms and worsen prognosis (139). Afro-Caribbean patients are at greater risk for coercive intervention, diagnosis of psychosis, police referral, social deprivation, and poor access to general practitioners (128). Future research needs to address these mental health disparities and the social suffering that they perpetuate in these communities. Careful, sustained attention to these problems will promote long-term collective well-being, reduced barriers to care, and empowerment of Afro-Caribbean voices through humanist approaches like participatory research.

Beyond the advice that we offer at the level of daily clinical practice, we recognize that many needed changes cannot be implemented at the micro-level of individual interactions but require higher-order meso- and macro-level changes of systems, structures, professional orders, and society as a whole to offer better, more accessible, and sustainable care (140).

## Conclusion

Afro-Caribbean patients and families embody a rich, complex historical heritage, and a troubled past haunted by issues like racism, economic precarity, family fragmentation and marginal status in White dominant societies. All of these merit urgent consideration by the research community (63, 64, 141–143). Histories of enslavement and colonialism continue to exert influence on people of Afro-Caribbean heritage (73, 77), such that historical, sociocultural, and geopolitical lenses are needed to bring psychosis into focus by clarifying its psychosocial causes and how they lead to culturally mediated clinical presentations of psychotic disorder. By attending to these broader issues, clinicians will reduce bias, minimize misdiagnosis, improve therapeutic relationships, and optimize treatment (129). Afro-Caribbean people share a long history of inferior, and at times discriminatory, psychiatric care, and clinical attention to cultural mistrust will foster communication between patients, families, and clinicians. Furthermore, as most psychological research is conducted in Western, educated, industrialized, rich and democratic (WEIRD) societies (144), where psychotherapy values individualism; recognizing this fact is a first step toward the inclusion of collectivist approaches to mental health that will lay the foundation for culturally informed family and community interventions for Afro-Caribbean people (8). We hope that the themes identified in this paper – colonialism, adaptation, cultural mistrust, collectivism, and religion and spirituality – will act as a general road map for clinicians and researchers as they evaluate people from English-speaking Afro-Caribbean backgrounds. This template focuses on key issues to consider during discussions, interviews and report writing that will offer a more accurate, nuanced, and sensitive portrait of what is at stake and what may be influencing the production of symptoms and behaviors and fostering holistic recovery. Self-reflection and self-awareness are core aspects of clinical and research training that make possible the integration of Afro-Caribbean perspectives into mental health care, consistent with cultural competence and humility. Beyond change at the level of the individual clinician, institutional and higher-order action (structural and systemic) is needed to promote mental health equity as a priority and implement policy changes accordingly.

## Author contributions

SK: Conceptualization, Data curation, Formal analysis, Investigation, Methodology, Writing – review & editing, Writing – original draft. XY: Conceptualization, Data curation, Formal analysis, Investigation, Methodology, Writing – original draft, Writing – review & editing. GJ: Conceptualization, Data curation, Formal analysis, Investigation, Methodology, Writing – review & editing, Funding acquisition, Project administration, Resources, Software, Supervision, Validation, Visualization.
