# Supplementary material for: “Dem sey mi mad”: a scoping review of the attitudes and beliefs of English-speaking Afro-Caribbeans about psychosis
Source: Front Psychiatry. 2024 Aug 19;15:1385525. doi: 10.3389/fpsyt.2024.1385525 (PMC11366823; doi:10.3389/fpsyt.2024.1385525)
Supplement: Supplementary file 1 [file DataSheet_1.pdf]

Appendix

Search strategy

((TITLE-ABS-KEY (psychosis OR schizophrenia OR "mental disorder*" OR "mental illness*")) AND (TITLE-ABS-KEY (cultur* OR belief* OR attitude* OR explanat* OR causal* OR conceptual* OR perception* OR religio* OR "traditional heal*" OR holistic OR faith OR spiritual*))) AND ((TITLE-ABS-KEY ("antigua and barbuda" OR barbados OR "british virgin islands" OR grenada OR jamaica* OR "saint kitts and nevis" OR "saint lucia" OR "saint vincent and the grenadines" OR "trinidad and tobago") OR TITLE-ABS-KEY (caribbean* W/2 (black* OR african*)))))

Supplementary material: Additional references that were part of our analysis and informed our writing overall, but were not cited in text

1. Culture: National Library of Medicine - National Institutes of Health; [Available from: https://www.ncbi.nlm.nih.gov/mesh/68003469.

2. Ethnic groups: National Library of Medicine - National Institutes of Health; [Available from: https://www.ncbi.nlm.nih.gov/mesh/68005006.

3. Côte-des-Neiges–Notre-Dame-de-Grâce, Profils sociodémographiques: Ville de Montréal; 2016 [Available from: https://ville.montreal.qc.ca/portal/page?_pageid=6897,68087635&_dad=portal&_schema=PORTAL.

4. Abdirahman HA, Bah TT, Shrestha HL, Jacobsen KH. Bullying, Mental Health, and Parental Involvement among Adolescents in the Caribbean. West Indian Medical Journal. 2012;61(5):504-8.

5. Albarus N, Whitehorne-Smith P, Abel W. Perceived Susceptibility to Mental Disorders among Marijuana Smokers Attending a Tertiary Institution. West Indian Medical Journal. 2017.

6. Annoual P, Bibeau G, Marshall C, Sterlin C. Enslavement, colonialism, racism, identity and mental health: Developing a new service model for Canadians of African Descent. 2007.

7. Arthur CM, Hickling FW, Robertson-Hickling H, Haynes-Robinson T, Abel W, Whitley R. "Mad, Sick, Head Nuh Good": Mental Illness Stigma in Jamaican Communities. Transcultural Psychiatry. 2010;47(2):252-75.

8. Ayonrinde OA. Schizophrenia-like psychosis in African and Caribbean elders. British Journal of Psychiatry. 2002;180(2):187-.

9. Baboolal N, Davis G, Stewart R, Ramesar J, McRae A. Comparisons between different elements of reported burden and common mental disorder in caregivers of ethnically diverse people with dementia in Trinidad. PLoS One. 2018;13(7):e0201165-e.

10. Barrowclough C, Tarrier N. Families of schizophrenic patients: Cognitive behavioural intervention. Boca Raton, FL: Chapman & Hall/CRC; 1992. xi, 263-xi, p.

11. Bennett J, Stennett R. Attitudes towards mental illness of nursing students in a Baccalaureate programme in Jamaica: a questionnaire survey. Journal of Psychiatric and Mental Health Nursing. 2015;22(8):599-605.

12. Bhattacharya R, Cross S, Bhugra D. Schizophrenia in African–Caribbeans: Contributing factors. 2010. In: Clinical Topics in Cultural Psychiatry [Internet]. London: The Royal College of Psychiatrists. Available from: http://www.SLQ.eblib.com.au/patron/FullRecord.aspx?p=521950.

13. Bhugra D. Ideas of Distorted Ethnic Identity in 43 Cases of Psychosis. International Journal of Social Psychiatry. 2001;47(1):1-7.

14. Bhugra D. Self-concept: Psychosis and attraction of new religious movements. Mental Health, Religion & Culture. 2002;5(3):239-52.

15. Bhugra D, Hilwig M, Hossein B, Marceau H, Neehall J, Leff J, et al. First-Contact Incidence Rates of Schizophrenia in Trinidad and One-Year Follow-up. British Journal of Psychiatry. 1996;169(5):587-92.

16. Bhugra D, Hilwig M, Mallett R, Corridan B, Leff J, Neehall J, et al. Factors in the onset of schizophrenia:a comparison between London andTrinidad samples. Acta Psychiatrica Scandinavica. 2000;101(2):135-41.

17. Bhugra RMJLD. Schizophrenia and African-Caribbeans: a conceptual model of aetiology. International Review of Psychiatry. 1999;11(2-3):145-52.

18. Bhui K, Mohamud S, Warfa N, Craig TJ, Stansfeld SA. Cultural adaptation of mental health measures: Improving the quality of clinical practice and research. British Journal of Psychiatry. 2003;183(3):184-6.

19. Boneham MA, Williams KE, Copeland JRM, McKibbin P, Wilson K, Scott A, et al. Elderly people from ethnic minorities in Liverpool: mental illness, unmet need and barriers to service use. Health & Social Care in the Community. 2007;5(3):173-80.

20. Burke AW. Epidemiological Aspects of the Repatriate Syndrome. International Journal of Social Psychiatry. 1982;28(4):291-9.

21. Burke AW. Outcome of Mental Illness Following Repatriation: a Predictive Study. International Journal of Social Psychiatry. 1983;29(1):3-11.

22. Cadge C, Connor C, Greenfield S. University students' understanding and perceptions of schizophrenia in the UK: a qualitative study. BMJ Open. 2019;9(4):e025813-e.

23. Campbell C, Cornish F, McLean C. Social Capital, Participation and the Perpetuation of Health Inequalities: Obstacles to African-Caribbean Participation in 'Partnerships' to Improve Mental Health. Ethn Health. 2004;9(3):305-27.

24. Cartwright SA. Report on the diseases and physical peculiarities of the Negro race. New Orleans, Louisiana: New Orleans Medical and Surgical Journal; 1851.

25. Chakraborty AT, McKenzie K, Leavey G, King M. Measuring perceived racism and psychosis in African-Caribbean patients in the United Kingdom: the modified perceived racism scale. Clinical practice and epidemiology in mental health: CP & EMH. 2009;5:10-.

26. Charalambides M, Morgan C, Murray RM. Epidemiology of Migration and Serious Mental Illness: The Example of Migrants to Europe. Textbook of Psychiatric Epidemiology: Wiley; 2011. p. 579-94.

27. Cheng TC, Robinson MA. Factors Leading African Americans and Black Caribbeans to Use Social Work Services for Treating Mental and Substance Use Disorders. Health & Social Work. 2013;38(2):99-109.

28. Chorlton E, McKenzie K, Morgan C, Doody G. Course and outcome of psychosis in black Caribbean populations and other ethnic groups living in the UK: A systematic review. International Journal of Social Psychiatry. 2011;58(4):400-8.

29. Codjoe L, Byrne M, Lister M, McGuire P, Valmaggia L. Exploring Perceptions of "Wellness" in Black Ethnic Minority Individuals at Risk of Developing Psychosis. Behavioural and Cognitive Psychotherapy. 2012;41(2):144-61.

30. Cohen AS, Mohr C, Ettinger U, Chan RCK, Park S. Schizotypy as an organizing framework for social and affective sciences. Schizophr Bull. 2015;41 Suppl 2(Suppl 2):S427-S35.

31. Das-Munshi J, Becares L, Dewey ME, Stansfeld SA, Prince MJ. Understanding the effect of ethnic density on mental health: multi-level investigation of survey data from England. BMJ. 2010;341:c5367-c.

32. Degnan A, Shattock L, Edge D. Cultural variations in attachment and psychosis. Attachment Theory and Psychosis: Routledge; 2019. p. 192-220.

33. Donaghay-Spire EG, McGowan J, Griffiths K, Barazzone N. Exploring narratives of psychological input in the acute inpatient setting. Psychology and Psychotherapy: Theory, Research and Practice. 2015;89(4):464-82.

34. Earl TR, Fortuna LR, Gao S, Williams DR, Neighbors H, Takeuchi D, et al. An exploration of how psychotic-like symptoms are experienced, endorsed, and understood from the National Latino and Asian American Study and National Survey of American Life. Ethn Health. 2015;20(3):273-92.

35. Eaton W, Harrison G. Ethnic disadvantage and schizophrenia. Acta Psychiatrica Scandinavica. 2000;102(s407):38-43.

36. Edge D. "Why are you cast down, o my soul?" Exploring intersections of ethnicity, gender, depression, spirituality and implications for Black British Caribbean women's mental health. Critical Public Health. 2013;23(1):39-48.

37. Emmanuel M, Campbell M. Commentary: A Comparative Review of Involuntary Admission of People with Mental Illness in China and Barbados. Journal of the American Academy of Psychiatry and the Law Online. 2015;43(1):45.

38. Erving CL. Physical-psychiatric comorbidity: patterns and explanations for ethnic group differences. Ethn Health. 2017;23(6):583-610.

39. Fernando S. Institutional Racism in Psychiatry and Clinical Psychology: Springer International Publishing; 2017.

40. Ferrari M, Flora N, Anderson KK, Haughton A, Tuck A, Archie S, et al. Gender differences in pathways to care for early psychosis. Early Intervention in Psychiatry. 2016;12(3):355-61.

41. Flora N, Barbaree H, Simpson AIF, Noh S, McKenzie K. Pathways to Forensic Mental Health Care in Toronto: A Comparison of European, African-Caribbean, and other Ethnoracial Groups in Toronto. The Canadian Journal of Psychiatry. 2012;57(7):414-21.

42. Fung WLA, Bhugra D, Jones PB. Ethnicity and mental health: the example of schizophrenia in migrant populations across Europe. Psychiatry. 2006;5(11):396-401.

43. Garraway H, Pistrang N. "Brother from another mother": Mentoring for African-Caribbean adolescent boys. Journal of Adolescence. 2010;33(5):719-29.

44. Gaston GB, Earl TR, Nisanci A, Glomb B. Perception of mental health services among Black Americans. Social Work in Mental Health. 2016;14(6):676-95.

45. Gibson RC, Abel WD, White S, Hickling FW. Internalizing stigma associated with mental illness: findings from a general population survey in Jamaica. Revista Panamericana de Salud Pública. 2008;23(1).

46. Gibson RC, Martin Js Fau - Neita SM, Neita SM. Mental illness and public health: exploring the role of general hospital physicians at a teaching hospital in Jamaica. West Indian Med J. 2010(0043-3144 (Print)).

47. Gilvarry CM, Walsh E, Samele C, Hutchinson G, Mallett R, Rabe-Hesketh S, et al. Life events, ethnicity and perceptions of discrimination in patients with severe mental illness. Social Psychiatry and Psychiatric Epidemiology. 1999;34(11):600-8.

48. Griffith EE. The impact of culture and religion on psychiatric care. J Natl Med Assoc. 1982(0027-9684 (Print)).

49. Griffiths S. A programme for changing attitudes in the statutory sector : dialogue is critical. In: Fernando S, Keating F, editors. Mental health in a multi-ethnic society : a multidisciplinary handbook. 2nd ed. London: Routledge; 2009.

50. Harding T. Marihuana-Modified Mania. Archives of General Psychiatry. 1973;29(5):635.

51. Harrison G, Amin S, Singh SP, Croudace T, Jones P. Outcome of psychosis in people of African–Caribbean family origin. British Journal of Psychiatry. 1999;175(1):43-9.

52. Hickling FW. The epidemiology of schizophrenia and other common mental health disorders in the English-speaking Caribbean. Revista Panamericana de Salud Pública. 2005;18(4-5):256-62.

53. Hickling FW. Understanding patients in multicultural settings: a personal reflection on ethnicity and culture in clinical practice. Ethn Health. 2012;17(1-2):203-16.

54. Hickling FW, Gibson RC, Hutchinson G. Current research on transcultural psychiatry in the Anglophone Caribbean: Epistemological, public policy, and epidemiological challenges. Transcultural Psychiatry. 2013;50(6):858-75.

55. Hickling FW, Hutchinson G. Post-colonialism and mental health. Psychiatric Bulletin. 2000;24(3):94-5.

56. Hickling FW, Hutchinson G. Caribbean Contributions to Contemporary Psychiatric Psychopathology. West Indian Medical Journal. 2012;61(4):442-6.

57. Hickling FW, Robertson-Hickling H Fau - Paisley V, Paisley V. Deinstitutionalization and attitudes toward mental illness in Jamaica: a qualitative study. Rev Panam Salud Publica. 2011(1680-5348 (Electronic)).

58. Hickling FW, Rodgers-Johnson P. The Incidence of First Contact Schizophrenia in Jamaica. British Journal of Psychiatry. 1995;167(2):193-6.

59. Hodes M, Vila GD, Kan C, Tolmac J, Kramer T. The mental health of British African-Caribbean children and adolescents. In: Garralda ME, Raynaud J-P, editors. Culture and conflict in child and adolescent mental health. IACAPAP book series. The working with children and adolescents series; v. 17. Lanham, Md.: Jason Aronson; 2008.

60. Holttum S. Research watch: people of Black African and African Caribbean heritage in the UK and USA – psychosis, racism and inclusion. Mental Health and Social Inclusion. 2020;24(2):67-74.

61. Hope MO, Assari S, Cole-Lewis YC, Caldwell CH. Religious Social Support, Discrimination, and Psychiatric Disorders among Black Adolescents. Race Soc Probl. 2017;9(2):102-14.

62. Horst H, Miller D. From Kinship to Link-up. Current Anthropology. 2005;46(5):755-78.

63. Huggins C. Predictors of Mental Health Treatment Utilization among African American and Caribbean

Black Older Adults [Doctoral dissertation]: New York University; 2013.

64. Hutchinson G, Takei N, Fahy TA, Bhugra D, Gilvarry C, Moran P, et al. Morbid Risk of Schizophrenia in First-Degree Relatives of White and African–Caribbean Patients with Psychosis. British Journal of Psychiatry. 1996;169(6):776-80.

65. Hyppolite J. The role of acculturation, racial identity and ethnic identity in understanding utilization rates, attitudes toward formal mental health services and preferences for help among Caribbean Black college students [Doctoral dissertation]. Brooklyn, New York: Long Island University; 2012.

66. Jackson D, Heatherington L. Young Jamaicans' attitudes toward mental illness: Experimental and demographic factors associated with social distance and stigmatizing opinions. Journal of Community Psychology. 2006;34(5):563-76.

67. Jackson DN. Attitudes toward seeking professional psychological help among Jamaican adolescents [Doctoral dissertation]: University of Miami; 2006.

68. Jackson Williams D. Where do Jamaican Adolescents Turn for Psychological Help? Child & Youth Care Forum. 2012;41(5):461-77.

69. Jackson Williams D. Help-Seeking Among Jamaican Adolescents. Journal of Black Psychology. 2013;40(4):359-83.

70. James CCAB, Peltzer K. Traditional and alternative therapy for mental illness in Jamaica: patients' conceptions and practitioners' attitudes. Afr J Tradit Complement Altern Med. 2011;9(1):94-104.

71. Keating F. African and Caribbean men and mental health. Ethnicity and Inequalities in Health and Social Care. 2009;2(2):41-53.

72. King M, Nazroo J, Weich S, McKenzie K, Bhui K, Karlson S, et al. Psychotic symptoms in the general population of England. Social Psychiatry and Psychiatric Epidemiology. 2005;40(5):375-81.

73. Kirkbride JB, Errazuriz A, Croudace TJ, Morgan C, Jackson D, Boydell J, et al. Incidence of schizophrenia and other psychoses in England, 1950-2009: a systematic review and meta-analyses. PLoS One. 2012;7(3):e31660-e.

74. Kirkbride JB, Jones PB, Ullrich S, Coid JW. Social deprivation, inequality, and the neighborhood-level incidence of psychotic syndromes in East London. Schizophr Bull. 2014;40(1):169-80.

75. Kirmayer LJ, Groleau D, Guzder J, Blake C, Jarvis E. Cultural Consultation: A Model of Mental Health Service for Multicultural Societies. The Canadian Journal of Psychiatry. 2003;48(3):145-53.

76. Knight S, Jarvis GE, Ryder AG, Lashley M, Rousseau C. ‘It Just Feels Like an Invasion’: Black First-Episode Psychosis Patients’ Experiences With Coercive Intervention and Its Influence on Help-Seeking Behaviours. Journal of Black Psychology. 2022;49(2):200-35.

77. Kogan CS, Noorishad P-G, Ndengeyingoma A, Guerrier M, Cénat JM. Prevalence and correlates of anxiety symptoms among Black people in Canada: A significant role for everyday racial discrimination and racial microaggressions. Journal of Affective Disorders. 2022;308:545-53.

78. Krishnakumar A, Narine L, Roopnarine JL, Logie C. Sociodemographic, psychosocial and physical health correlates of common mental disorder symptoms among mothers in Trinidad and Tobago: Examining ethnic variations. International Journal of Psychology. 2016;53(4):304-12.

79. Lacey KK, Sears KP, Govia IO, Forsythe-Brown I, Matusko N, Jackson JS. Substance use, mental disorders and physical health of Caribbeans at-home compared to those residing in the United States. Int J Environ Res Public Health. 2015;12(1):710-34.

80. Lawrence V, Murray J, Banerjee S, Turner S, Sangha K, Byng R, et al. Concepts and Causation of Depression: A Cross-Cultural Study of the Beliefs of Older Adults. The Gerontologist. 2006;46(1):23-32.

81. Leach M. Jamaican Duppy Lore. The Journal of American Folklore. 1961;74(293):207.

82. LeBihan N. Working with Families of African Caribbean Origin: Understanding Issues around Immigration and Attachment by Elaine Arnold. Canadian Art Therapy Association Journal. 2012;25(1):25-.

83. Littlewood R. From vice to madness: The semantics of naturalistic and personalistic understandings in Trinidadian local medicine. Social Science & Medicine. 1988;27(2):129-48.

84. Lloyd KR, Jacob KS, Patel V, St. Louis L, Bhugra D, Mann AH. The development of the Short Explanatory Model Interview (SEMI) and its use among primary-care attenders with common mental disorders. Psychological Medicine. 1998;28(5):1231-7.

85. Mahy GE, Mallett R, Leff J, Bhugra D. First-contact incidence rate of schizophrenia on Barbados. British Journal of Psychiatry. 1999;175(1):28-33.

86. Mallett R, Leff J, Bhugra D, Pang D, Zhao JH. Social environment, ethnicity and schizophrenia. Social Psychiatry and Psychiatric Epidemiology. 2002;37(7):329-35.

87. Mallett R, Leff J, Bhugra D, Takei N, Corridan B. Ethnicity, Goal Striving and Schizophrenia: A Case-Control Study of Three Ethnic Groups in the United Kingdom. International Journal of Social Psychiatry. 2004;50(4):331-44.

88. Maloney CA, Abel WD, McLeod HJ. Jamaican adolescents' receptiveness to digital mental health services: A cross-sectional survey from rural and urban communities. Internet Interv. 2020;21:100325-.

89. Manna V, Daniele MT, Pinto M. Ethnopsychopharmacology. Biological and cultural aspects of response to medical treatment in psychiatry. Italian Journal of Psychopathology. 2010;16:326-45.

90. Maraj A, Iyer SN, Shah JL. Enhancing the Engagement of Immigrant and Ethnocultural Minority Clients in Canadian Early Intervention Services for Psychosis. Can J Psychiatry. 2018;63(11):740-7.

91. Marques S, Godinho F, Melo AL, Barrocas D. First-episode psychosis: What does it mean? European Psychiatry. 2016;33(S1):s258-s.

92. Marwaha S, Livingston G. Stigma, racism or choice. Why do depressed ethnic elders avoid psychiatrists? Journal of Affective Disorders. 2002;72(3):257-65.

93. Mathur R, Hull SA, Boomla K, Robson J. Ethnic differences in primary care management of diabetes and cardiovascular disease in people with serious mental illness. The British journal of general practice: the journal of the Royal College of General Practitioners. 2012;62(601):e582-e8.

94. Matthews CJ. Generational status, racial, and ethnic identity, and psychological well-being among Afro-Caribbean college students [Doctoral dissertation]: Fordham University; 2012.

95. McIntyre JC, Elahi A, Barlow FK, White RG, Bentall RP. The relationship between ingroup identity and Paranoid ideation among people from African and African Caribbean backgrounds. Psychology and Psychotherapy: Theory, Research and Practice. 2019;94(1):16-32.

96. McKenzie K, Jones P, Lewis S, Williams M, Toone B, Sham P, et al. Lower prevalence of pre-morbid neurological illness in African-Caribbean than White psychotic patients in England. Psychological Medicine. 2002;32(7):1285-91.

97. McLean C, Campbell C, Cornish F. African-Caribbean interactions with mental health services in the UK: experiences and expectations of exclusion as (re)productive of health inequalities. Social Science & Medicine. 2003;56(3):657-69.

98. Mistry H, Osborn D. Underuse of clozapine in treatment-resistant schizophrenia. Advances in Psychiatric Treatment. 2011;17(4):250-5.

99. Mohan R, McCrone P, Szmukler G, Micali N, Afuwape S, Thornicroft G. Ethnic Differences in Mental Health Service Use Among Patients with Psychotic Disorders. Social Psychiatry and Psychiatric Epidemiology. 2006;41(10):771-6.

100. Monk EP. Linked fate and mental health among African Americans. Social Science & Medicine. 2020;266:113340.

101. Morgan C, Fearon P. Social experience and psychosis. Insights from studies of migrant and ethnic minority groups. Epidemiologia e Psichiatria Sociale. 2007;16(2):118-23.

102. Morgan C, Fearon P, Lappin J, Heslin M, Donoghue K, Lomas B, et al. Ethnicity and long-term course and outcome of psychotic disorders in a UK sample: the ÆSOP-10 study. The British journal of psychiatry: the journal of mental science. 2017;211(2):88-94.

103. Morgan C, Hibben M, Esan O, John S, Patel V, Weiss HA, et al. Searching for psychosis: INTREPID (1): systems for detecting untreated and first-episode cases of psychosis in diverse settings. Social psychiatry and psychiatric epidemiology. 2015;50(6):879-93.

104. Morgan C, John S, Esan O, Hibben M, Patel V, Weiss H, et al. The incidence of psychoses in diverse settings, INTREPID (2): a feasibility study in India, Nigeria, and Trinidad. Psychological Medicine. 2016;46(9):1923-33.

105. Morgan C, Mallett R, Hutchinson G, Leff J. Negative pathways to psychiatric care and ethnicity: the bridge between social science and psychiatry. Social Science & Medicine. 2004;58(4):739-52.

106. Murray RM, Fearon P. Searching for racists under the psychiatric bed: Commentary on … Institutional racism in psychiatry. Psychiatric Bulletin. 2007;31(10):365-6.

107. Nazroo J, Iley K. Ethnicity, migration and mental health: the role of social and economic inequalities. Migration and Mental Health: Cambridge University Press; 2010. p. 79-97.

108. Nazroo JY, Bhui KS, Rhodes J. Where next for understanding race/ethnic inequalities in severe mental illness? Structural, interpersonal and institutional racism. Sociol Health Illn. 2020;42(2):262-76.

109. Nicolas G, Schwartz B. Culture first: Lessons learned about the importance of the cultural adaptation of cognitive behavior treatment interventions for Black Caribbean youth. Cultural adaptations: Tools for evidence-based practice with diverse populations: American Psychological Association; 2012. p. 71-90.

110. Oliver EA, Fearon P. Schizophrenia: epidemiology and risk factors. Psychiatry. 2008;7(10):410-4.

111. Oluwatayo O, Gater R. The role of engagement with services in compulsory admission of African/Caribbean patients. Social Psychiatry and Psychiatric Epidemiology. 2004;39(9):739-43.

112. Parasram HDMR. The practice of psychiatry in Trinidad and Tobago. International Review of Psychiatry. 1999;11(2-3):173-83.

113. Payne JAM. An exploration of the attitudes toward and beliefs about mental health and mental health seeking behaviors among Jamaican immigrants living in the United States [Doctoral dissertation]. Washington, D. C.: Catholic University of America; 2006.

114. Perry CM. “You can’t speak Creole in here. English only”: Experiences of Stigma and Acts of Resistance among Adults of Haitian Descent in the Bahamas. International Journal of Bahamian Studies. 2020;26:61.

115. Phoenix A. Colourism and the Politics of Beauty. Feminist Review. 2014;108(1):97-105.

116. Pinto R, Ashworth M, Jones R. Schizophrenia in black Caribbeans living in the UK: an exploration of underlying causes of the high incidence rate. Br J Gen Pract. 2008;58(551):429-34.

117. Pusey-Murray A, Miller P. 'I need help': caregivers' experiences of caring for their relatives with mental illness in Jamaica. Ment Health Fam Med. 2013(1756-834X (Print)).

118. Rabiee F, Smith P. Understanding mental health and experience of accessing services among African and African Caribbean Service users and carers in Birmingham, UK. Diversity and Equality in Health and Care. 2014;11.

119. Radua J, Ramella-Cravaro V, Ioannidis JPA, Reichenberg A, Phiphopthatsanee N, Amir T, et al. What causes psychosis? An umbrella review of risk and protective factors. World psychiatry : official journal of the World Psychiatric Association (WPA). 2018;17(1):49-66.

120. Ramkissoon AK, Donald C, Hutchinson G. Supernatural versus medical: Responses to mental illness from undergraduate university students in Trinidad. International Journal of Social Psychiatry. 2017;63(4):330-8.

121. Ratan DA. Schizophrenia in Trinidad. British Journal of Psychiatry. 1997;170(4):389-.

122. Rathod S, Kingdon D, Phiri P, Gobbi M. Developing Culturally Sensitive Cognitive Behaviour Therapy for Psychosis for Ethnic Minority Patients by Exploration and Incorporation of Service Users' and Health Professionals' Views and Opinions. Behavioural and Cognitive Psychotherapy. 2010;38(5):511-33.

123. Rathod S, Kingdon D, Pinninti N, Turkington D, Phiri P. Cultural Adaptation of CBT for Serious Mental Illness: Wiley; 2015 2015/02/17.

124. Rathod S, Phiri P, Harris S, Underwood C, Thagadur M, Padmanabi U, et al. Cognitive behaviour therapy for psychosis can be adapted for minority ethnic groups: A randomised controlled trial. Schizophrenia Research. 2013;143(2-3):319-26.

125. Roberts N. Bouffée Délirante in Jamaican Adolescent Siblings. The Canadian Journal of Psychiatry. 1990;35(3):251-3.

126. Röhricht F, Basdekis-Jozsa R, Sidhu J, Mukhtar A, Suzuki I, Priebe S. The association of religiosity, spirituality, and ethnic background with ego-pathology in acute schizophrenia. Mental Health, Religion & Culture. 2009;12(6):515-26.

127. Rüdell K, Bhui K, Priebe S. Do 'alternative' help-seeking strategies affect primary care service use? A survey of help-seeking for mental distress. BMC Public Health. 2008;8:207-.

128. Sanchez D, Awad GH. Ethnic group differences in racial identity attitudes, perceived discrimination and mental health outcomes in African American, Black Caribbean and Latino Caribbean college students. International Journal of Culture and Mental Health. 2015;9(1):31-43.

129. Schofield P, Kordowicz M, Pennycooke E, Armstrong D. Ethnic differences in psychosis-Lay epidemiology explanations. Health Expect. 2019;22(5):965-73.

130. Scior K, Potts HW, Furnham AF. Awareness of schizophrenia and intellectual disability and stigma across ethnic groups in the UK. Psychiatry Research. 2013;208(2):125-30.

131. Secker J, Harding C. African and African Caribbean users' perceptions of inpatient services. Journal of Psychiatric and Mental Health Nursing. 2002;9(2):161-7.

132. Selten J-P, Cantor-Graae E, Kahn RS. Migration and schizophrenia. Current Opinion in Psychiatry. 2007;20(2):111-5.

133. Selten J-P, Hoek HW. Does misdiagnosis explain the schizophrenia epidemic among immigrants from developing countries to Western Europe? Social Psychiatry and Psychiatric Epidemiology. 2008;43(12):937-9.

134. Selten J-P, van Os J, Cantor-Graae E. The social defeat hypothesis of schizophrenia: issues of measurement and reverse causality. World psychiatry : official journal of the World Psychiatric Association (WPA). 2016;15(3):294-5.

135. Shah A. The 'Count Me In' psychiatric in-patient census for 2007 and the elderly: evidence of improvement or cause for concern? Psychiatric Bulletin. 2009;33(6):201-3.

136. Sharpley M, Hutchinson G, Murray RM, McKenzie K. Understanding the excess of psychosis among the African-Caribbean population in England. British Journal of Psychiatry. 2001;178(S40):s60-s8.

137. Sharpley MS, Peters ER. Ethnicity, class and schizotypy. Social Psychiatry and Psychiatric Epidemiology. 1999;34(10):507-12.

138. Shenton AK. Strategies for ensuring trustworthiness in qualitative research projects. Education for Information. 2004;22(2):63-75.

139. Shim RS, Ye J Fau - Baltrus P, Baltrus P Fau - Fry-Johnson Y, Fry-Johnson Y Fau - Daniels E, Daniels E Fau - Rust G, Rust G. Racial/ethnic disparities, social support, and depression: examining a social determinant of mental health. Ethn Dis. 2012(1049-510X (Print)).

140. Sobo EJ. The Jamaican body's role in emotional experience and sense perception: Feelings, hearts, minds, and nerves. Cult Med Psychiatry. 1996;20(3):313-42.

141. Stone L, Finlay WML. A Comparison of African-Caribbean and White European Young Adults' Conceptions of Schizophrenia Symptoms and the Diagnostic Label. International Journal of Social Psychiatry. 2008;54(3):242-61.

142. Swinnen SGHA, Selten J-P. Mood disorders and migration. British Journal of Psychiatry. 2007;190(1):6-10.

143. Thachil A, Bhugra D. Globalization and Mental Health – Traditional Medicine in Pathways to Care in the United Kingdom. Psychiatrists and Traditional Healers: Wiley; 2009. p. 215-28.

144. Thornicroft G, Farrelly S, Birchwood M, Marshall M, Szmukler G, Waheed W, et al. CRIMSON [CRisis plan IMpact: Subjective and Objective coercion and eNgagement] protocol: a randomised controlled trial of joint crisis plans to reduce compulsory treatment of people with psychosis. Trials. 2010;11:102-.

145. Tortelli A, Errazuriz A, Croudace T, Morgan C, Murray RM, Jones PB, et al. Schizophrenia and other psychotic disorders in Caribbean-born migrants and their descendants in England: systematic review and meta-analysis of incidence rates, 1950-2013. Social psychiatry and psychiatric epidemiology. 2015;50(7):1039-55.

146. Turner N, Hastings JF, Neighbors HW. Mental health care treatment seeking among African Americans and Caribbean Blacks: what is the role of religiosity/spirituality? Aging Ment Health. 2019;23(7):905-11.

147. Venner H, Welfare LE. Black Caribbean Immigrants: A Qualitative Study of Experiences in Mental Health Therapy. Journal of Black Psychology. 2019;45(8):639-60.

148. Watt D. Traditional Religious Practices amongst African-Caribbean Mothers and Community Othermothers. Black Theology. 2004;2(2):195-212.

149. Whaley AL. Help-seeking from indigenous healers among persons of African ancestry in the United States: Ethnic and racial disparities in mental and physical health. Complementary Therapies in Medicine. 2019;45:222-7.

150. Whaley AL. Associations between seeking help from indigenous healers and symptoms of depression versus psychosis in the African diaspora of the United States. Counselling and Psychotherapy Research. 2020;21(1):179-87.

151. Winer RA, Morris-Patterson A, Smart Y, Bijan I, Katz CL. Knowledge of and Attitudes Toward Mental Illness Among Primary Care Providers in Saint Vincent and the Grenadines. Psychiatric Quarterly. 2013;84(3):395-406.

152. Winkler AC. The duppy. Brooklyn, NY: Akashic Books; 2013. Available from: https://search.ebscohost.com/login.aspx?direct=true&scope=site&db=nlebk&db=nlabk&AN=944815.

153. Woodward AT, Bullard KM, Taylor RJ, Chatters LM, Baser RE, Perron BE, et al. Complementary and alternative medicine for mental disorders among African Americans, black Caribbeans, and whites. Psychiatric services. 2009;60(10):1342-9.

154. Yorke CB. Jamaican immigrants: Help seeking attitudes toward mental health care [Doctoral dissertation]: Columbia University; 2006.

155. Youssef FF, Bachew R, Bodie D, Leach R, Morris K, Sherma G. Knowledge and attitudes towards mental illness among college students: Insights into the wider English-speaking Caribbean population. International Journal of Social Psychiatry. 2012;60(1):47-54.
